# Editorial: Uncovering the relationship between myelodysplastic syndromes and acute myeloid leukemia

**DOI:** 10.3389/fonc.2023.1131785

**Published:** 2023-01-20

**Authors:** Mohamed A. Yassin, Ahmed Elsabagh

**Affiliations:** ^1^ Hematology Section, Medical Oncology, National Center for Cancer Care and Research (NCCCR), Hamad Medical Corporation (HMC), Doha, Qatar; ^2^ College of Medicine, Qatar University (QU), Doha, Qatar; ^3^ College of Medicine, Qatar University (QU) Health, Qatar University, Doha, Qatar

**Keywords:** myelodysplastic/myeloproliferative disorders, AML – acute myeloid leukemia, genetic, pathology, progress

Myelodysplastic syndromes (MDSs) have a 5-year transformation risk to AML of 20%. These cases are also associated with poor outcomes. Hence, this highlights the importance of uncovering the underlying triggers and pathophysiology of this progression to find ways to hinder this process. In addition, both conditions should be distinguished clearly to maximize the benefit of treatment.

Wong et.al investigated the effect of hypomethylating agents (HMAs), specifically decitabine and guadecitabine, in acute myeloid leukemia (AML) and myelodysplastic syndromes (MDS). They discussed the role of HMAs in increasing the expression of cancer/testis antigens (CTAs), which are a subgroup of tumor-associated antigens (TAAs), in tumors. CTAs increase immune responses when they are expressed outside their usual locations in immune-privileged organs. This leads to an increased antileukemic response in MDS and AML. The CTA NY-ESO-1 is one of the most studied CTAs and is highly increased in those tumors. When this method is combined with cancer vaccines, which utilize immunologic activity against CTAs, the immunogenicity and response would be stronger and more effective since more antigen presentation would be present on tumor cells. Evidence has shown that adding guadecitabine to vaccines may increase the capability of cytotoxic T-cells (CTLs) to kill tumor cells. There is also evidence that combining HMAs with immune checkpoint inhibitors, such as programmed cell death (PD-1)/programmed death ligand-1 (PDL-1) inhibitors, enhances the response to therapy as it may overcome resistance. Low-dose decitabine was also shown to trigger natural killer (NK) cell response against AML antigens. This still requires further studies as well as clinical trials to confirm the predicted effects of HMAs on AML and MDS patients.

Joudinaud and Boyer investigated the mechanisms causing the progression of MDSs to AML. The origins of these cells were also discussed. They showed that MDS cells are derived from hematopoietic stem cells that may acquire genetic aberrations. Leukemic stem cells, on the other hand, are derived from progenitor cells. The review demonstrates the rise in CD123 and CLL1 markers on both leukemic and myelodysplastic syndrome stem cells. CD45RA was also shown to be an interesting marker for leukemic stem cells since they can be combined with CD34, CD38, and CD90 to approximate the cells found at the time of diagnosis. They also compared the most common genetic aberrations in MDS compared to AML. In AML, mutations in genes encode tyrosine kinase receptors in the RAS pathway. In MDS, there are more mutations involving epigenetic regulators and splicing proteins. The emergence of specific somatic mutations in high-risk MDS patients was found to be related to faster progression to AML. These mutations are non-linear since MDS stem cells gain those mutations in parallel. In their review of the metabolic activity of the malignant stem cells, they found that MDS stem cells demonstrate higher utilization of the citric acid cycle and oxidative phosphorylation. Hence, targeting these processes and hindering them acts as a possible method for treatment.

Moreover, Jawad et al. used data mining and databases to assess the effect of DNMT3A with arginine (R)882 mutations. They concluded that MDS with DNMT3A R882 mutations are associated with a higher number of blasts and increased risk of progression to AML when compared to non-R882 mutations. They demonstrated that a variety of clinical outcomes can result from DNMT3A mutations according to the type of mutation. R882 mutations lead to a markedly reduced methyltransferase activity which leads to an increased leukemogenic potential. However, DNMT3A was reduced in clonal hematopoiesis of indeterminate potential (CHIP) and MDS. The R882 mutation in MDS was a major prognostic indicator for severe leukopenia and reduced progression-free survival (PFS). It was also linked with a higher risk of progression to AML when compared to non-R882 mutations. This shows that selective inhibition of R882, allowing the wild-type DNMT3A to function, could be a possible treatment option. They also show that the risk of AML transformation in R882 mutations is dependent on SF3B1 and SRSF2 mutations since these mutations reduce the rate of transformation to zero in some studies.

In addition, Al-Bulushi et al. studied the impact of epigenetic mutations on prognosis and overall survival (OS) in AML. They concluded that ASXL1, TET2, DNMT3A, and IDH mutations have a negative effect on OS in AML patients. They found IDH to be linked with intermediate-risk patients (according to the international prognostic scoring system), elderly patients, and patients with higher white blood cell (WBC) counts. Nevertheless, many studies included in the meta-analysis had several limitations and showed different results which show that more well-performed clinical research and data are necessary to confirm these findings.

Finally, Ambinder and DeZern compared the characteristics of MDS and AML that may affect the prognosis and management of the diseases. MDS differs from AML as it shows a bone marrow blast percentage of less than 20% and no AML-defining genetic mutations. Nevertheless, this cutoff to diagnose AML may not always be effective in therapy, since some MDS patients may gain an increased benefit from AML-type treatment. Venetoclax-based therapy exhibited adequate safety and efficacy in high-risk MDS. They also discuss the importance of differentiating secondary AML from *de novo* AML since secondary AML is usually associated with poorer clinical outcomes. The presence of residual MDS cells with AML cells complicates treatment further since both should be treated simultaneously.

## Author contributions

Both authors listed have made a substantial, direct, and intellectual contribution to the work and approved it for publication.

